# Non-Cardiac Surgery in Developing Countries: Epidemiological Aspects and Economical Opportunities – The Case of Brazil

**DOI:** 10.1371/journal.pone.0010607

**Published:** 2010-05-12

**Authors:** Pai Ching Yu, Daniela Calderaro, Danielle Menosi Gualandro, Andre Coelho Marques, Adriana Feio Pastana, Joao Carlos Prandini, Bruno Caramelli

**Affiliations:** 1 Interdisciplinary Medicine in Cardiology Unit, Heart Institute (InCor) – Hospital das Clínicas - University of São Paulo Medical School, São Paulo, Brazil; 2 Mathematics Department, University of São Paulo, Sao Paulo, Brazil; University of Oxford, United Kingdom

## Abstract

**Background:**

Worldwide distribution of surgical interventions is unequal. Developed countries account for the majority of surgeries and information about non-cardiac operations in developing countries is scarce. The purpose of our study was to describe the epidemiological data of non-cardiac surgeries performed in Brazil in the last years.

**Methods and Findings:**

This is a retrospective cohort study that investigated the time window from 1995 to 2007. We collected information from DATASUS, a national public health system database. The following variables were studied: number of surgeries, in-hospital expenses, blood transfusion related costs, length of stay and case fatality rates. The results were presented as sum, average and percentage. The trend analysis was performed by linear regression model. There were 32,659,513 non-cardiac surgeries performed in Brazil in thirteen years. An increment of 20.42% was observed in the number of surgeries in this period and nowadays nearly 3 million operations are performed annually. The cost of these procedures has increased tremendously in the last years. The increment of surgical cost was almost 200%. The total expenses related to surgical hospitalizations were more than $10 billion in all these years. The yearly cost of surgical procedures to public health system was more than $1.27 billion for all surgical hospitalizations, and in average, U$445.24 per surgical procedure. The total cost of blood transfusion was near $98 million in all years and annually approximately $10 million were spent in perioperative transfusion. The surgical mortality had an increment of 31.11% in the period. Actually, in 2007, the surgical mortality in Brazil was 1.77%. All the variables had a significant increment along the studied period: r square (r^2^) = 0.447 for the number of surgeries (P = 0.012), r^2^ = 0.439 for in-hospital expenses (P = 0.014) and r^2^ = 0.907 for surgical mortality (P = 0.0055).

**Conclusion:**

The volume of surgical procedures has increased substantially in Brazil through the past years. The expenditure related to these procedures and its mortality has also increased as the number of operations. Better planning of public health resource and strategies of investment are needed to supply the crescent demand of surgery in Brazil.

## Introduction

It was recently estimated that every year 234.2 millions of surgeries are performed around the world. The authors, however, argued that this number is probably underestimated because of incomplete data and unregistered interventions [1]. Moreover, the authors observed that worldwide distribution of surgical interventions is unequal. Developed countries account for the majority of surgeries and information about non-cardiac operations in developing countries is scarce. Indeed, in developed countries surgical procedures are associated to mortality rates ranging from 0.4 to 0.8% and morbidity from 3 to 16% whereas in developing countries there is a completely different picture: mortality rate ranges from 5 to 10% [1].

Along the last decades, several strategies have been suggested to be effective in the reduction of perioperative complications [2]–[18]. **Table 1**. However, in spite of all medical and technological innovation, surgical mortality and morbidity remain above desirable levels and, more importantly, there is a huge worldwide variation suggesting that application of standards of evidence regarding to perioperative care is not adequate in some countries.

**Table 1:** Effective strategies to reduce perioperative complications.

Perioperative evaluation/risk estimation based on clinical algorithms and guidelines [2]–[4]
Beta-blockade in special clinical settings [5]–[10]
Statins for patients submitted to vascular surgery [11]–[14]
Clinical and cardiological monitorization in ICU for high risk patients [2], [15]
Intensive glucose control [16], [17]
Surveillance with cardiac biomarkers (cardiac troponin) [15], [18]

In the last decade, economical growth has significantly changed lifestyle and characteristics of the population of developing countries. The increase of life expectancy associated to the changes in demographic characteristics of population, besides the technological and medical advances, may have resulted in modifications on the number of surgeries, surgical mortality, number of days of in-hospital stay and health expenditure. The purpose of our study was to evaluate the epidemiological and economical aspects of non-cardiac surgeries performed in Brazil in the last years.

## Methods

This is a retrospective study that investigated national data about a cohort of individuals submitted to non-cardiac surgery in Brazil in the last years. The study was designed and conducted at the Heart Institute from the University of Sao Paulo, Brazil. The data were collected from DATASUS (www.datasus.gov.br), a national open-access public health system database, organized and maintained by the government. We selected surgical hospitalizations information from DATASUS based on its relevance and its potential contribution to provide epidemiological information regarding non-cardiac surgeries performed in Brazil. The variables studied were: number of surgeries, in-hospital expenses, blood transfusion related cost, period of hospitalization (length of stay) and case-fatality rates.

We accessed DATASUS database on January of 2009 and collected information of all medical hospitalizations. In DATASUS, every hospitalization is described by the corresponding name of procedure and a respective code number. The database contained 3,209 types of procedures related to all areas of medicine, 785 of them were classified as non-surgical hospitalizations and were excluded from our analysis. Surgical interventions not in the focus of this study, such as cardiac surgery, ambulatory surgery, minor surgery (eg.: diagnostic procedures), biopsy and birth delivery were excluded as well. Occasionally, a surgical procedure was found in duplicate in the database with the same name and a different code number. In this case, the information of repeated procedures was grouped into a single category. After that, 1,568 non-cardiac surgeries were selected and constituted the database of the present study.

In our original database, the selected information was available from 1992 to 2007, but we decided to exclude data previous to 1995 because of the different monetary system used before this date in Brazil, which jeopardized the comparison of economical data between these periods. The final time-window of our registry was from 1995 to 2007.

### Ethics Statement

This study was conducted according to the principles expressed in the Declaration of Helsinki. The study was approved by the Institutional Review Board of Hospital das Clinicas - University of Sao Paulo Medical School (number 0952/09). Since all the information of the study was collected from an open-access database, inform consent was not obtained in our study due to no potential patient identifiers in dataset.

### Statistical analysis

For each studied variable, we obtained a single dataset where the information was represented with the corresponding year in column and surgical procedure in row. We found a significant number of missing data in the original database provided by DATASUS. The surgical procedures containing missing data were not included in the statistical analysis. A descriptive statistical analysis was performed and the results were presented as sum, average and percentage. The trend analysis was performed by linear regression model. All the statistical analyses were performed by the statistical program R – project for statistical computing and SPSS version 17.0. We adopted as statistical significance, value of P<0.05 for all the tests performed.

The economic data showing in-hospital expenses related to surgical hospitalizations were analyzed after correction of its values to monetary depreciation in the period. For this purpose, we used for correction the national economic index, specific for health costs. The value of all expenses were converted from Real to US dollar 2009 (The conversion rate was R$1.76 for $1.00 at the time of the study).

## Results

We analyzed 32,659,513 non-cardiac surgeries performed in Brazil during thirteen years. From 1995 to 2007 we observed an increment of 20.42% in the number of surgical procedures. After adjustment for the population growth, we observed a consistent increase in the number of surgeries per 1,000 inhabitants in the studied period, remarkably from 2001 to 2007. In addition, we observed also a progressive increase in case-fatality rates and in the expenses related to surgical interventions. **Table 2**.

**Table 2:** Results of studied variables and its changes among the time-window from 1995 to 2007.

Year	Number of Surgeries	Number of surgeries/1,000 inhabitants	Total in-hospital expenses (U$)	Blood transfusion cost (U$)	Length of stay (days)	Mortality
1995	2,374,785	14.95	430,863,966.29	4,854,739.24	4.36	1.35%
1996	2,240,815	13.89	437,971,979.33	4,770,430.97	4.46	1.46%
1997	2,152,741	13.14	434,812,544.63	5,153,020.23	4.37	1.53%
1998	2,205,637	13.27	520,001,042.93	6,263,305.91	4.34	1.54%
1999	2,359,313	13.98	674,983,936.25	7,274,983.84	4.31	1.57%
2000	2,505,951	14.63	698,072,190.81	7,591,026.47	4.07	1.53%
2001	2,412,859	13.88	731,001,821.51	7,805,656.50	4.16	1.61%
2002	2,578,830	14.63	824,119,175.40	7,809,363.30	4.12	1.58%
2003	2,672,604	14.95	936,352,419.05	7,743,472.55	4.06	1.65%
2004	2,748,461	15.18	1,063,584,652.73	9,036,363.77	3.99	1.68%
2005	2,750,971	15.00	1,140,509,016.19	10,375,029.49	3.91	1.78%
2006	2,796,745	15.07	1,158,672,620.45	9,953,549.40	3.82	1.74%
2007	2,859,801	15.24	1,273,316,507.84	10,140,843.08	3.83	1.77%
**Total**	32,659,513		10,324,261,873.41	98,771,784.73		

We observed an increment of total in-hospital expenses as well as blood transfusion related costs in association with surgical interventions. The total in-hospital expenses related to surgical hospitalizations had an increment nearly to 200% in a period of 13 years reaching the comprehensive value of more than $10 billions of dollars in all these years (more than $1.27 billions of dollars per year). In average, $ 445.24 dollars were spent per patient per surgical procedure. Similarly, the same pattern of increment was observed in expenditures related to perioperative blood transfusions. There was an increment in the transfusional cost of more than 100% in the period and the entire cost related to blood transfusion was almost $ 98 millions of dollars in all these years. **Table 2**.

The case-fatality rates related to these non-cardiac surgeries increased 31.11% during the last thirteen years. The length of stay was the single variable that reduced through the last years. In average, the length of stay had a decline of 12.15%. **Table 2**.

### Trends analysis

**Figure 1:**
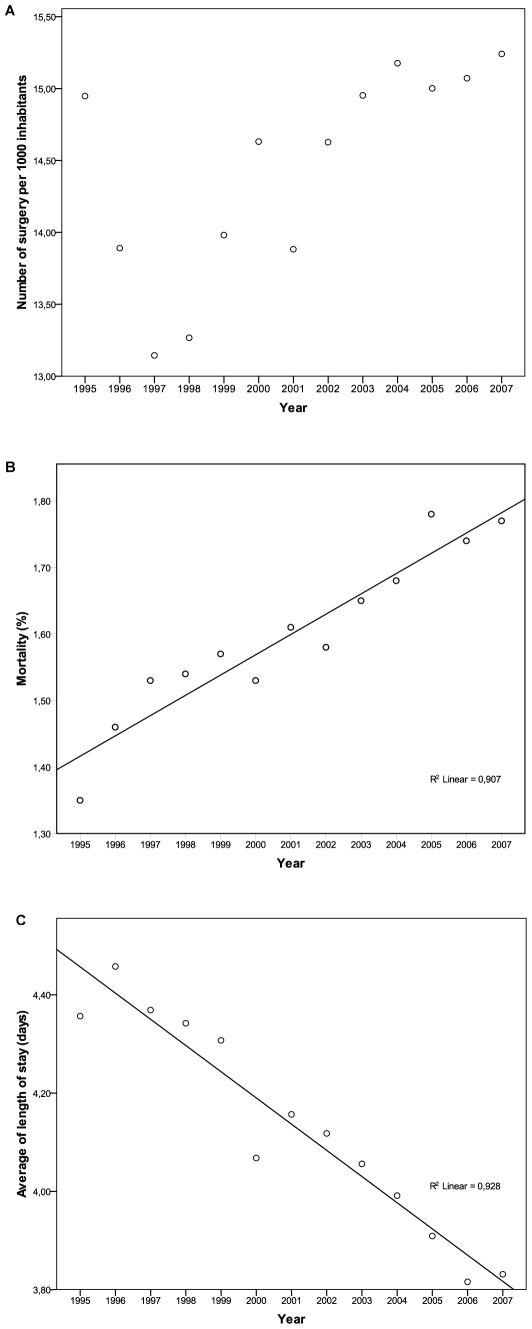
Trends analysis results. A- Number of surgery per 1,000 inhabitants. B – Mortality. C- Length of Stay.

Globally, the trend analysis showed that the number of surgeries per 1,000 inhabitants and the surgical mortality had a statistically significant increment along the past thirteen years (r^2^ = 0.447, P = 0.012 and r^2^ = 0.907, P = 0.005, respectively). On the other hand, the length of stay had a significant decline in the period (r^2^ = 0.928, P = 0.001). **Figure 1**.

### Economical analysis adjusted for monetary depreciation

For the economical analysis of expenses related to surgical hospitalizations, we used the average cost of the surgical procedures. All the values were corrected to monetary depreciation that occurred in the studied period. For this correction, we used the national economic index, specific for health costs. The year of 1995 was used as monetary reference. Generally, there was a reduction of surgical expenses per procedure in the first two years, in 1996 and 1997, as observed in our database. After that, we observed a progressive increase of surgical expenses through the last years. **Table 3**.

**Table 3:** Economical data of surgical procedures performed from 1995 to 2007 adjusted for monetary depreciation.

Year	Mean hospital expense per surgery (U$)	Mean transfusion cost per surgery (U$)
1995	181.43	2.04
1996	132.44	1.44
1997	115.83	1.37
1998	127.88	1.54
1999	150.33	1.62
2000	142.37	1.55
2001	154.24	1.65
2002	160.56	1.52
2003	169.89	1.40
2004	178.48	1.52
2005	182.25	1.66
2006	176.57	1.52
2007	184.99	1.47

The values were corrected to monetary depreciation in the period. The year 1995 was used as reference.

**Figure 2:**
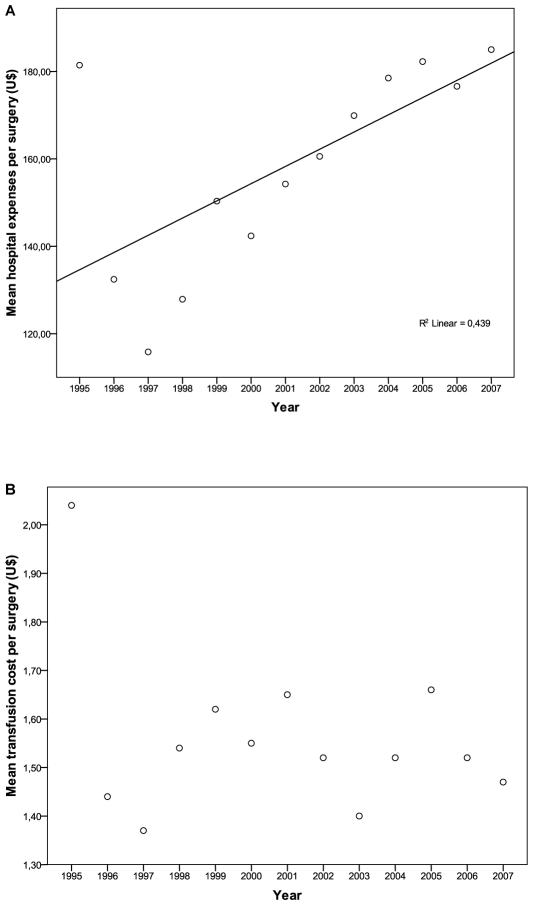
Trend analysis of economical data adjusted for monetary depreciation. A – Mean in-hospital expense per surgery. B- Mean transfusion cost per surgery.

The trend analysis of surgical hospitalization expenses was statistically significant and demonstrated a progressive increase of in-hospital expenses associated to surgical procedures (r^2^ = 0.439, P = 0.014). Regarding the increment of blood transfusion expenses observed during the studied period, it disappeared after adjustment for monetary depreciation. (r^2^ = 0.014, P = 0.30). **Figure 2**.

## Discussion

Our results indicated that the volume of non-cardiac surgeries performed in Brazil has increased substantially through the past years. In parallel, the increase of the number of the operations implicated in the elevation of total hospital expenses related to these surgical procedures. The surgical mortality on our registry has also increased along these years. According to our findings, the epidemiology of surgeries performed in Brazil is different from international data. Annually, there are performed almost 38 millions of surgeries in United States of America, and 7 millions of surgeries are estimated to be performed annually in Europe. In our registry, Brazil performed nearly 3 millions of non-cardiac surgeries yearly, a number much lower compared to developed countries. These numbers may indicate unequal access to surgical treatment between these countries.

In a previous study of the estimation of the global volume of surgery, Weiser et al [1] described a disparity in access to surgery between rich and poor countries and that there is a close relation between per-head expenditure on health and country's annual surgical rate (r^2^ = 0.868, P<0.0001). Countries with middle ($401–1,000) and high-expenditures per capita ($>1,000) on health care were responsible for 73.6% of surgical procedures worldwide, whereas the countries with poor (<$100) and low-expenditures ($101–400) provided only 26.5% of operations but accounted for 69.8% of the global population.

In the last decades, Brazil showed a great economical development that was paralleled by a significant modification in the health characteristics of the population. There was a reduction in perinatal mortality and an increase in life expectancy [19]. In spite of the increment of life expectancy, investment of the government in the public health system in Brazil remains below desired levels. The analysis of health expenditure ratios in Brazil demonstrated that total expenditure on health as percentage of Brazilian gross domestic product showed an increase since the last report in 2000 [19]. However, regardless of the increment from 7.2% in 2000 to 7.5% in 2006, it remains below the global rate of 8.7%. In addition, the public expenditure on health was responsible for only 48% of total expenditure on health, corresponding to only 7.2% of total public expenditure, significantly lower than the global value of 14.3%. The total expenditure on health per capita per year, in Brazil is, in average, $ 427. From this amount, only $ 204 were invested by the government, a value much inferior when compared to global expenditure of $ 429 per capita or to the investment from developed countries like United States of America ($ 3,076 per capita) or Europe ($ 1,350 per capita) [19]. In addition to our data, these numbers indicate that the public investment on health in Brazil is significantly lower than international patterns.

On the other hand, the results of our registry suggested that surgical procedures in Brazil are associated to higher mortality rate and lower hospital expenditure per operation when compared to developed countries. In average, the mean value spent per surgical procedure was $ 445.24 in Brazil, whereas surgical hospital expenses accounts for more than $ 2,000 per operation in a canadian institution [20] or superior to $ 9,000 in an american institution [21]. Regarding to case-fatality rates, we found that the perioperative mortality in Brazil is two times greater than american rates [21] and almost nine times higher than canadian rates [20]. The findings related to the length of stay in our registry are comparable to international data.

According to the categories established by Weiser and cols in their study [1], Brazil would be classified as a middle-expenditure country with an estimated rate of surgery of 42.48 operations per 1,000 inhabitants. However, in our study, despite the non inclusion of cardiac surgeries, minor interventions and birth deliveries, we found that only 15.24 surgical procedures were performed annually per 1,000 inhabitants. The results of our study in conjunction with these numbers suggest that, in Brazil, the access to surgical interventions is deficient, there is an insufficient number of surgeries performed, and the surgical outcome is worse. This picture requires more attention and investment to obtain better results.

### Study limitations

Limited information is available regarding non-cardiac surgery outcomes, the overall number of operations and surgical morbidity and mortality. To our knowledge, this is the pioneer registry designed to evaluate epidemiological national data about non-cardiac surgeries. The major limitation of our study is that it is a retrospective study based on information collected from an administrative database. The use of data based on administrative information is susceptible to potential sources of bias. The feed of information into dataset is usually performed by administrative technicians and non-medical professionals and the system is susceptible to typing errors, wrong data interpretation or missing data. We sought to exclude the missing data for the analysis in order to minimize the errors of the results. Another limitation is that the time-window of our study was only thirteen years and it was impossible to make future estimations.

However, this is the first national registry about non-cardiac surgeries that provided important information about epidemiological and economical aspects of operations performed in Brazil. The crescent increment in the number of surgeries associated to the increasing of global life expectancy may increase substantially the burden of surgical interventions in the next years. This elevated number of operations performed annually and its high mortality has emerged as a global public-health challenge. The knowledge of epidemiological data of surgeries performed in each country and its progression over the years is essential to define strategies and priorities in public health policy. Our data may contribute to this debate and also help to a better planning of public health investment in Brazil.

### Conclusion

The volume of surgical procedures has increased substantially in Brazil through the past years. The expenditure related to these procedures and its mortality has also increased as the number of operations. Better planning of public health resource and strategies of investment are needed to supply the crescent demand of surgery in Brazil.
